# Retraction: Evaluate teaching quality of physical education using a hybrid multi-criteria decision-making framework

**DOI:** 10.1371/journal.pone.0325685

**Published:** 2025-06-03

**Authors:** The PLOS One Editors

The *PLOS One* Editors retract this article [1] due to concerns about reliance on retracted work cited in References 4, 5, and 12.

SL did not agree with the retraction. ZC either did not respond directly or could not be reached.
